# Efficient Genome Editing Using CRISPR/Cas9 Technology in Chicory

**DOI:** 10.3390/ijms20051155

**Published:** 2019-03-06

**Authors:** Guillaume Bernard, David Gagneul, Harmony Alves Dos Santos, Audrey Etienne, Jean-Louis Hilbert, Caroline Rambaud

**Affiliations:** EA 7394, Institut Charles Viollette (ICV) Agro-food and Biotechnology Research Institute, Université de Lille, INRA, ISA, Univ. Artois, Univ. Littoral Côte d’Opale, Cité Scientifique, 59655 Villeneuve d’Ascq, France; guillaume.bernard04000@gmail.com (G.B.); david.gagneul@univ-lille.fr (D.G.); harmony.alves-dos-santos@univ-lille.fr (H.A.D.S.); audrey.etienne@univ-lille.fr (A.E.); jean-louis.hilbert@univ-lille.fr (J.-L.H.)

**Keywords:** CRISPR/Cas9, multiplex genome editing, *Cichorium intybus*, phytoene desaturase, *Agrobacterium rhizogenes-*mediated transformation, protoplast transformation

## Abstract

CRISPR/Cas9 (Clustered Regularly Interspaced Short Palindromic Repeats/CRISPR associated with protein CAS9) is a genome-editing tool that has been extensively used in the last five years because of its novelty, affordability, and feasibility. This technology has been developed in many plant species for gene function analysis and crop improvement but has never been used in chicory (*Cichorium intybus* L.). In this study, we successfully applied CRISPR/Cas9-mediated targeted mutagenesis to chicory using *Agrobacterium rhizogenes*-mediated transformation and protoplast transfection methods. A *U6* promoter (*CiU6*-1p) among eight predicted *U6* promoters in chicory was selected to drive sgRNA expression. A binary vector designed to induce targeted mutations in the fifth exon of the chicory *phytoene desaturase* gene *(CiPDS)* was then constructed and used to transform chicory. The mutation frequency was 4.5% with the protoplast transient expression system and 31.25% with *A. rhizogenes*-mediated stable transformation. Biallelic mutations were detected in all the mutant plants. The use of *A. rhizogenes*-mediated transformation seems preferable as the regeneration of plants is faster and the mutation frequency was shown to be higher. With both transformation methods, foreign DNA was integrated in the plant genome. Hence, selection of vector (transgene)-free segregants is required. Our results showed that genome editing with CRISPR/Cas9 system can be efficiently used with chicory, which should facilitate and accelerate genetic improvement and functional biology.

## 1. Introduction

Genome editing, which consists of targeting and digesting DNA at a specific site in the genome, is an important tool for gene function analysis and crop improvement [1]. To date, three specific genome-editing technologies have been developed: zinc finger nucleases (ZFNs) [2], transcription activator-like effector nucleases (TALENs) [3], and clustered regularly interspaced short palindromic repeat (CRISPR) associated protein (CAS) system. These methods induce double strand-breaks (DSBs) in the targeted DNA. In eukaryotic cells, these breaks can be repaired in two different pathways: the non-homologous end-joining (NHEJ) and homologous recombination (HR). NHEJ is the most commonly used DSB repair mechanism in many organisms and can cause insertions or deletions that can potentially produce a gene knockout [4].

In natural conditions, the CRISPR/Cas9 system uses a CRISPR RNA (crRNA) and a small trans-activating CRISPR RNA (trancrRNA), which can hybridize to form the mature dual crRNA. The CAS9 endonuclease forms a ribonucleoprotein complex with the crRNA, which guides the endonuclease to a specific target DNA. Cleavage is only possible if the complex recognizes a short seed sequence upstream of a protospacer-adjacent motif (PAM) 5′-NGG-3′ [5]. Thanks to molecular biology engineering, crRNA has been replaced by a chimeric single guide RNA (sgRNA). Consequently, only sgRNA and endonuclease CAS9 are needed to cut and create DSBs at specific loci in the genome of the cells. Because of its simplicity, low cost, versatility, and high efficiency, the CRISPR/Cas9 system has become the most widely used technology for genome editing in many organisms such as bacteria, yeasts, animals, and plants.

In plants, CRISPR/Cas9 system is often inserted in a vector containing the *CAS9* upstream of a strong ubiquitous promoter such as ubiquitin or Cauliflower Mosaic Virus (CaMV) 35S promoters. Moreover, germ-line specific promoters, such as *CDC45,* which have the advantage of being active in the meiotic/zygotic phase, have recently been successfully used [6]. To deliver the CAS9 protein to the nuclear genome, a nuclear localization signal (NLS) sequence is fused to the *CAS9* gene. To express the sgRNA, RNA polymerase-III promoters such as *U6* and *U3* are used. The transcript initiation nucleotide is G and A in the *U6* promoter and in the *U3* promoter, respectively [7]. Using Golden Gate cloning technology, which uses second-generation restriction enzymes [8], it is possible to easily insert numerous sgRNA and the *CAS9* gene in a single expression vector, in order to simultaneously target several DNA sites [9].

To introduce vectors into plants, two main transformation methods are commonly used in plants: protoplast transformation, where plasmids can be delivered into protoplasts using various methods, such as polyethylene glycol (PEG) fusion, electroporation and microinjection; and leaf tissue transformation using *Agrobacterium tumefaciens* or *Agrobacterium rhizogenes (Rhizobium rhizogenes)*. Both methods have been successfully applied to express CRISPR/Cas9 system in plants for genome editing.

Protoplasts have been used in Arabidopsis and tobacco [10] but also in crop species such as maize [11], rice, wheat [12], soybean [13] and tomato [14]. However, the regeneration of plants from mutated protoplasts is uncommon because many plant species cannot be regenerated from protoplast cultures, particularly the monocots. In addition, the regeneration process is long and subject to contamination. This approach is therefore often used as a preliminary step in evaluating the functionality of the CRISPR/Cas9 system before using it with a more appropriate transformation method such as *A. tumefaciens*-mediated transformation [15].

However, not all plant species can be transformed by these methods. In this case, other techniques may be used such as biolistic or *A. rhizogenes*-mediated transformation. *A. rhizogenes* has been widely used for plant transformation [16] to study rhizosphere or metabolic pathways. Genome editing using *A. rhizogenes*-mediated transformation has been successfully carried out in tomato (*Solanum spp*) [17], soybean (*Glycine max)* [13], *Brassica carinata* [18], *Salvia miltiorrhiza* [19]. However only a few plants have the ability to regenerate from hairy root lines [16], which hampers its use in genome editing.

Chicory (*Cichorium intybus L*.), an Asteraceae, is well known in traditional medicine. More than 100 individual compounds have been isolated from this plant, especially from its roots which are rich in specialized metabolites (also known as secondary metabolites or plant natural products) such as polyphenols and terpenoids [20,21,22]. Due to their ability to produce valuable compounds, hairy roots and cell cultures have been set up by biotechnologists [23]. It has been reported that specialized metabolites present in chicory exert many biological activities such as antioxidant, anti-cancer, anti-inflammatory or anti-hepatotoxic activities with beneficial effects on health in humans and livestock [24]. Chicory is also used as a vegetable crop, for leaf salad called chicon or witloof or as a coffee substitute. In addition, chicory roots have an inulin content which can be up to 40%. Inulin is a polysaccharide used as a sugar substitute due to its low caloric value [25]. Chicory is used for multiple applications. This has led to large scale cultivation and therefore to an interest in genetically modified chicory to improve yields and nutritional properties and thus extend its potential [26]. Targeted mutagenesis using CRISPR/Cas9 technology could potentially be used in chicory as an efficient tool for studying gene function and generating new varieties.

The success of targeted mutagenesis with CRISPR/Cas9 method depends on the efficiency of the different molecular components (sgRNA design, CAS9, promoters...) but mainly on the ability of the species to be transformed and to regenerate plants. *A. tumefaciens-*mediated transformation of chicory is feasible but genotype dependent [27], whereas its transformation using *A. rhizogenes* is quite efficient and the regeneration of plants from hairy roots is possible [28,29]. This may provide a fast approach for chicory genome editing. This strategy has already been used in *Taraxacum kok-saghyz* [30]. Otherwise PEG-mediated protoplast fusion has already been applied to induce male sterility in chicory and fused protoplasts are able to regenerate [31]. The use of protoplasts for genome editing could be an alternative in the case of chicory.

In this study, we performed an efficient multiplex genome editing in chicory in the first generation (T0), using two sgRNAs and CAS9. Both *A. rhizogenes*-mediated stable transformation and protoplast transient expression system were successfully applied to engineer chicory genome. As a proof of principle, we succeeded in knocking out the *CiPDS* gene (*phytoene desaturase*) in plants regenerated from hairy roots and from protoplasts. With *CiPDS* specific sgRNAs, several independent albino lines were generated. An efficient method that can be used for chicory mutagenesis is thus provided.

## 2. Results

### 2.1. Prediction of U6 Promoter Sequences in Chicory

**Figure 1:**
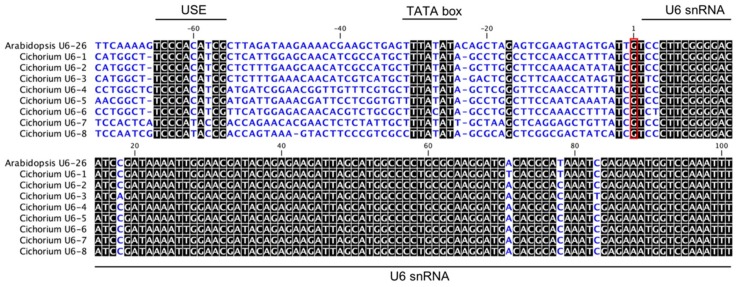
Chicory U6 and Arabidopsis U6-26 gene sequence partial alignment. The highly conserved parts of the sequences are in black while the Upstream Sequence Elements (USE), the TATA-boxes and the U6 small nuclear (snRNA) genes are marked with black bars. The transcription start sites are indicated (red frame).

The U6 RNA polymerase-III promoters are typically used to drive the sgRNA expression in Dicots [7]. The *Arabidopsis U6-26* promoter has been used to generate sgRNA in numerous plant species, such as in soybean [13], cotton [32], or *Salvia miltiorrhiza* [19]. To identify the *U6* promoters in chicory, the *Arabidopsis U6-26* small nuclear RNA (snRNA) sequence was used to search the chicory genome (unpublished database from Florimond Desprez SA). Eight U6 sequences (named *U6-1* to *U6-8*) with the highest similarity to the Arabidopsis sequence were selected for further analysis (Figure 1).

Polymerase-III promoters express transcripts with purine, i.e., a G, as the initiation nucleotide. As expected, a G was located just in front of all chicory snRNA sequences. All snRNA, including that of Arabidopsis, were highly similar. The *U6* promoter sequences contain two conserved elements essential for the transcription initiation by the RNA polymerase-III [33]: the upstream sequence element (USE; consensus sequence RTCCCACATCG) and a TATA-like box (consensus sequence TTTATATA). The USE sequence of *U6-7* (GenBank accession MK455778) and *U6-8* (GenBank accession MK455779) and the TATA-like box of *U6-6* (GenBank accession MK455777) are slightly different from the others. *U6-1* (GenBank accession MK455772), *U6-2* (GenBank accession MK455773), *U6-3* (GenBank accession MK455774), *U6-4* (GenBank accession MK455775) and *U6-5* (GenBank accession MK455776) could be good candidates for driving the expression of sgRNAs in chicory. *U6-1* promoter (*CiU6-1p*) was randomly chosen and used for the following experiments.

### 2.2. Target Selection and Plasmid Construction for the CRISPR/Cas9 System

**Figure 2:**
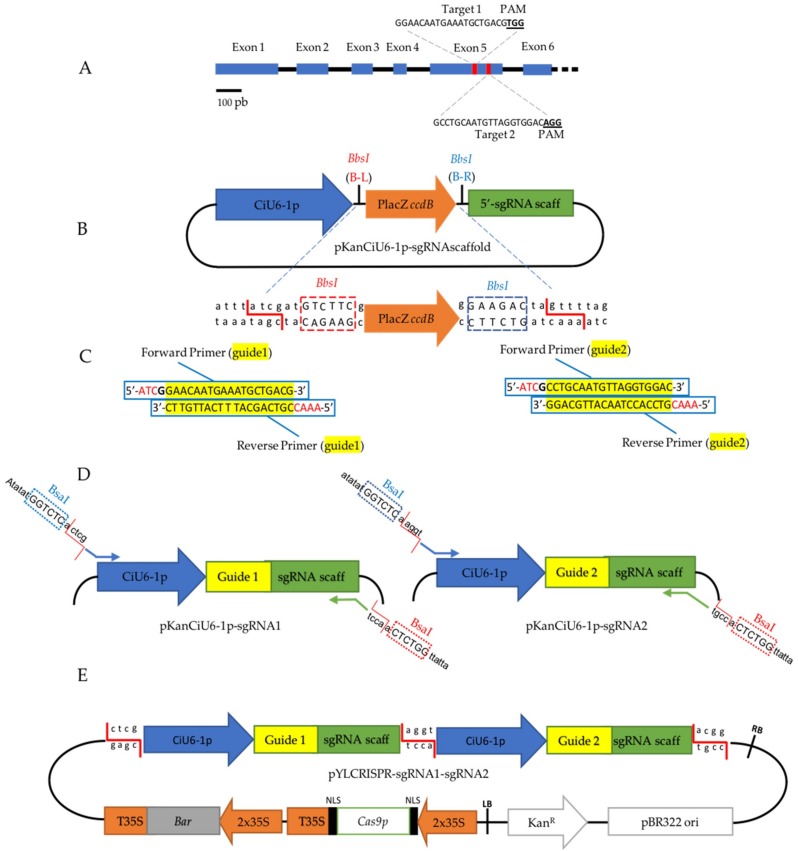
Overview of the experimental design for *CiPDS* disruption. (**A**) Schematic position of the two guide RNAs (red boxes) targeting the fifth exon of *CiPDS*. The blue boxes indicate exons; the black lines indicate introns. (**B**) Schematic view of pKanCiU6-1p-sgRNAscaffold. Between the *CiU6-1p* and the sgRNA scaffold (scaff), a *ccdB* gene driven by the LacZ promoter (counter selection marker) is surrounded by *BbsI* (second-generation enzyme) recognition sites, which allows the ligation of the hybridized guide adaptor shown in **C**. Red B-L depicts the orientation of the *BbsI* recognition site which achieves the cleavage on the left side. Blue B-R depicts the orientation of the *BbsI* recognition site which achieves cleavage on the right side. (**C**) Sequences of the complementary guide adaptors with the 19 pb guide sequence (yellow), the transcription initiator nucleotide G (bold), and the binding sites necessary to insert the hybridized guide adaptor into the pKanCiU6-1p-sgRNAscaffold (red). (**D**) Representation of the vector resulting from the insertion of the hybridized guide adaptors in the pKanCiU6-1p-sgRNAscaffold, (note that one vector is constructed for each guide). The primers used for preparation of CiU6-1p-guide-sgRNA cassettes for Golden Gate Cloning are also shown. (**E**) Representation of the final plasmid pYLCRISPR-sgRNA1-sgRNA2 resulting from the cloning of the two cassettes into the pYLCRISPR/Cas9P_35S_-B [36]. RB = Right Border, LB = Left Border, NLS = Nuclear Localization Signal.

A proof-of-principle experiment showed that *CiPDS* gene (*phytoene desaturase)* was successfully knocked out in plants regenerated from hairy roots and from protoplasts. PDS is a key enzyme for chlorophyll biosynthesis and *PDS* mutants are easily detectable due to their albino phenotype [34]. To identify putative *PDS* genes, a tBLASTn search of the chicory genome (unpublished database of Florimond Desprez SA) was conducted with *Helianthus annuus* (Asteraceae) PDS protein sequence (GenBank accession AHA36971.1) as a query. The gene with the highest similarity was selected. Only one copy of the gene was predicted. *CiPDS* (GenBank accession MK455771) is a 2514 pb in length gene and has 10 exons, with 1746 nucleotides of transcript sequence coding 581 amino acids. To knock out the gene, we designed 2 target sequences (double mutations) in the fifth exon (Figure 2A; Supplementary Figure S1). Using crispor.tefor.net software [35], 2 guide RNAs were selected according to the position of the target sequence and to their GC content. The CAS9 nuclease was expected to cut twice in exon 5 upstream of the PAM.

Our goal was to produce a binary vector harboring 2 sgRNAs and the *CAS9* for gene editing in chicory. The first step was to construct a plasmid with the *CiU6-1p* and the sgRNA scaffold, in which a guide RNA (gRNA) can be easily inserted. For this, we designed a sub-cloning vector containing the *CiU6-1p*, followed by a counter selection marker (lacZ-*ccdB, ccdB* under the control of the Isopropyl β-D-1-Thiogalactopyranoside (IPTG) inducible LacZ promoter) flanked by 2 *BbsI* recognition sites and the sgRNA scaffold (Figure 2C). This sequence was synthetized and inserted in pUCIDT-Kan to give pKanCiU6-1p-sgRNA. *BbsI* is a second-generation enzyme that produces distinct, non-palindromic sticky ends outside of its recognition site (Figure 2B). This feature is convenient in this case. Indeed, a G must be located directly upstream of the *CiU6-1p* to initiate the transcription and the gRNA must be positioned in front of the sgRNA scaffold. The 19 pb of the gRNAs with specific adaptors were synthetized as two complementary pairs of oligonucleotides (Table A1) as described by Ma and Liu [36] (Figure 2C) and inserted into pKanCiU6-1p-sgRNA by digestion/ligation to produce the plasmid pKanCiU6-sgRNA. For each guide, one plasmid was generated (pKanCiU6-1p-sgRNA1 and pKanCiU6-1p-sgRNA2; Figure 2D). The complete cassettes were amplified with specific primer pairs (GG1-F, GG1-R, and GG2-F, GG2-R) designed for Golden Gate cloning [8,37] and inserted into the pYLCRISPR/Cas9P_35S_-B expression vector [36]. Using this protocol, vector pYLCRISPR-sgRNA1-sgRNA2 (Figure 2E) engineered to co-express two sgRNAs and the CAS9 nuclease in chicory was constructed within a week.

### 2.3. Regeneration of CRISPR-Edited Plants From Protoplasts and From Hairy Roots Lines

**Figure 3:**
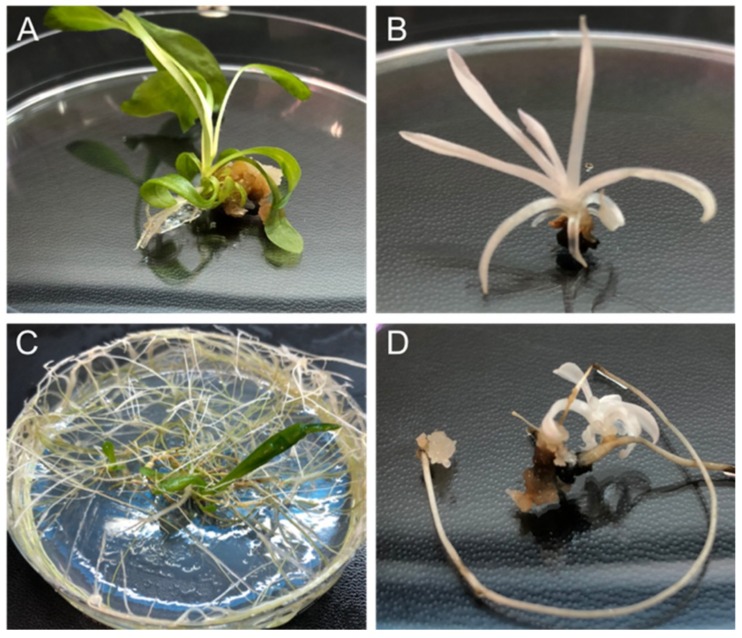
Phenotype of genome edited plants. (**A**) Wild-type (WT) shoot emerging from a WT callus. (**B**) *CiPDS* edited albino shoots emerging from a callus. (**C**) Hairy root line transformed with wild-type *A. rhizogenes* strain 15834 with emerging shoot. (**D**) Albino shoot emerging from hairy root line engineered to knock out *CiPDS*.

Two transformation methods were used to express the CRISPR/Cas9 system in chicory: protoplast transformation mediated with PEG and *A. rhizogenes*-mediated stable transformation. The protoplasts were transformed with the pYLCRISPR-sgRNA1-sgRNA2, transferred to growth media to obtain calli, and then plant regeneration was induced on a regeneration medium. As control, plants regenerated from protoplasts following the same protocols but without plasmid addition were obtained. Albino and green plants were obtained 5 months after transformation (Figure 3A,B). From 198 calli resulting from one transformation experiment, 9 calli developed albino shoots (4.5%, see below for genetic confirmation) (Table 1). These data show that it is possible to obtain mutated chicory plants from protoplasts transformed by PEG-mediated method.

Regarding the stable transformation with *A. rhizogenes* and to show that transformed hairy roots were able to regenerate plants with edited genomes, chicory was infected with *A. rhizogenes* strain 15834 transformed with the pYLCRISPR-sgRNA1-sgRNA2 binary plasmid or not (used as a control). Two weeks after *A. rhizogenes* transformation, 32 hairy root lines were generated from young leaves wounded with bacteria harboring the vector pYLCRISPR-sgRNA1-sgRNA2. The lines were cultivated on MS/2 medium with 300 mg/L ampicillin to remove the agrobacteria. From the 32 selected lines, 10 albino and 22 green lines (Figure 3C,D) were generated. These results were further confirmed at genetic level (see below). Non-albino lines grew more quickly than albino lines. From the 10 albino lines, 4 lines spontaneously regenerated albino plants. The others have regenerated albino plants after cultivation on bud induction medium MC4 typically used for chicory regeneration [38].

### 2.4. Identification of Mutation Events in Genome Edited Plants

To confirm the genome editing at the molecular level and to characterize the mutations, we extracted genomic DNA from all the different albino materials (calli, hairy roots, regenerated plants). The DNA from the hairy root lines was extracted before the regeneration of the first albino shoots. Chicory is a diploid species and genes have one copy in each homologous chromosome. Hence, the CRISPR/Cas9 system can induce two types of mutations in the targeted gene. On the one hand, only one of the alleles can be mutated, leading to a so-called monoallelic mutation. This type of mutation does not give a full knock out of the targeted gene because the other allele remains wild-type. On the other hand, the two alleles can be mutated which produces a biallelic mutation. In this case, if the two alleles share the same mutation, the biallelic mutation is homozygous, but if mutations are different, biallelic mutation is heterozygous.

**Figure 4:**
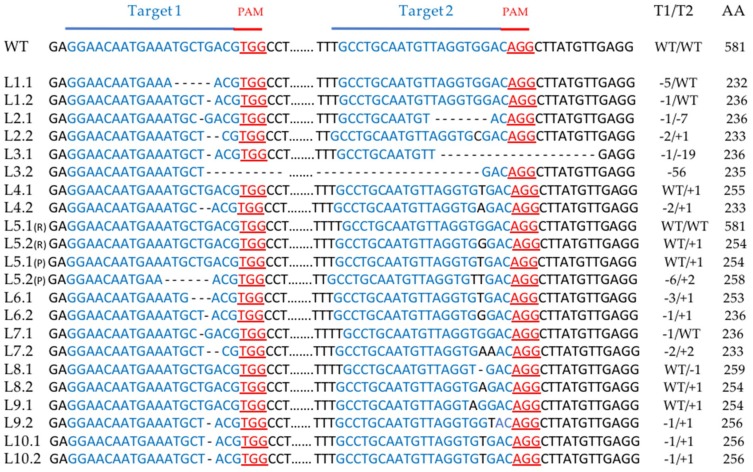
Sequence analysis of the two alleles of the 10 albino hairy root lines. The target sequences are depicted in blue and the PAM sequences in red. The change in the number of nucleotides is shown on the right of each allele sequence. A + indicates an insertion and a – stands for a deletion. WT indicates that there is no mutation. Example: -5/WT = deletion of 5 nucleotides on the first target site, no mutation on the second target site. For the L5: (R) = Hairy root stage sequence, (P) = plant stage sequence. The length of the amino acid chain in the truncated protein is shown on the right. Lx.x: line x allele x; AA: Amino Acid chain length; T1: Target1; T2: Target2.

**Figure 5:**
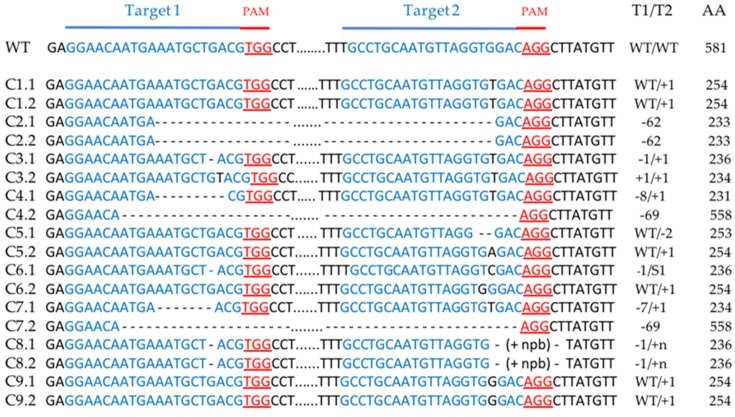
Sequence analysis of the two alleles of 9 albino calli obtained after protoplast transformation. The target sequences are depicted in blue and the PAM sequences in red. The change in the number of nucleotides is shown on the right of each allele sequence. A + or a – indicate an insertion or a deletion, respectively. A S means that there is a substitution. WT indicates that there is no mutation. Example: -5/WT = deletion of 5 nucleotides on the first target site, no mutation on the second target site. The length of the amino acid chain in the truncated protein is shown on the right. Cx.x: Callus x allele x; AA: Amino Acid chain length; T1: Target1; T2: Target2. (+ npb) = n base pairs resulting from insertion of plasmid parts.

To determine and characterize the different types of mutations obtained in our experiments, we amplified the *PDS* region including the two target sequences, using exon 5 specific primer pairs (E5-F and E5-R), and sequenced the polymerase chain reaction (PCR) products (around 420 bp). All types of mutations were detected. The monoallelic and heterozygous biallelic mutations producing superimposed sequence chromatograms from direct sequencing were decoded using Degenerate Sequence Decoding method [39]. Mutation analysis confirmed that all T0 albino plants contained mutant alleles in the *PDS* gene for at least one of the two targeted sites (Figure 4 and Figure 5). The mutation efficiency of the first target (target 1) differs between the two types of transformation: 66.6% with protoplast transformation and 90% with *A. rhizogenes*-mediated transformation. No differences were observed for target 2 (Table 1). Most of the mutation events were small insertions, (one or two bp) or small deletions (less than 19 pb) most probably because of the NHEJ repair mechanism (Figure 4 and Figure 5). However, in some cases, the inter-guide fragment was completely deleted, resulting in a larger deletion, as shown for L3 (Figure 4) and for C2, C4 and C7 (Figure 5). This inter-guide deletion can be detected before sequencing by electrophoresis in agarose gel, (Supplementary Figures S2 and S3). In all the albino plants analyzed, biallelic mutations responsible for gene knock out were found for at least one of the two target sites. In most cases, the insertion or deletion of a nucleotide in the target sequence induced an early termination of protein translation (Figure 4 and Figure 5, and Supplementary Figures S4 and S5). However, a deletion of the inter-guide fragment could just create a gap in the protein as shown for C4 and C7 (Supplementary Figure S5). In these examples, this gap is sufficient to induce the knock out of the gene.

Genetic analysis of either albino plants or the calli from which they originated showed that they shared the same mutation. No mosaic plants were found, showing that the mutation event was achieved at the protoplast stage. One hairy root line, the line L5 (Figure 4), did not carry the same mutation at hairy root stage than one month later at plant stage, suggesting that the CRISPR/Cas9 system may continue to modify the genes during the hairy root stage. This has been previously shown in soybean hairy roots [13]. All other albino plants have the same mutation than the hairy root line from which they originated, suggesting that mutations were stable in these lines.

All the lines analyzed gave a PCR product at the expected size, i.e., about 420 pb, except the C8 line (Supplementary Figure S3). PCR yielded a product of about 1500 pb, suggesting that during the repair mechanism, a large DNA fragment may also be inserted at the DNA cleavage site of the CAS9 protein. This phenomenon has already been observed in *Gossypium hirsutum* in which a large endogenous fragment (99 pb) had been inserted at the target site of the CAS9 protein [32]. Sequencing results of C8 line (Figure 5) showed that a DNA fragment was inserted at the target 2 site, in both alleles. This fragment is composed of multiple pYLCRISPR-sgRNA1-sgRNA2 fragments (Supplementary Figure S6).

### 2.5. Transgene Detection in Albino Plants

During the *A. rhizogenes*-mediated transformation, the T-DNA of the binary vector (pYLCRISPR-sgRNA1-sgRNA2) is inserted into the genome of the plant, as well as the T-DNA of the Ri (Root Inducing) plasmid containing the *rol* genes responsible for the hairy root phenotype. Indeed, due to the expression of the *virulence* (*vir)* genes present on the RI plasmid, the Right Border (RB) and Left Border (LB) of the two T-DNAs are recognized and the two T-DNAs are excised, transported, and integrated into the nuclear DNA and therefore a double transformation event occurs. PCRs with *rolB* gene specific primer pair and with *CAS9* specific primer pair showed that in all the albino plant lines regenerated from hairy root, the two T-DNAs were integrated into their genomes (data not shown). Further analysis was performed to determine whether mutated protoplast-derived plants had integrated the *CAS9* sequence. PCR with *CAS9*-specific primers showed that 3 plants out of 9 albino plants regenerated from protoplasts contained at least one fragment of the *CAS9* sequence (Supplementary Figure S7). This shows that vector fragments are inserted into the plant genome during the transformation procedure. Moreover, sequencing of the C8 plant, having inserted a fragment at the guide 2 target site, showed that this fragment was composed of many parts of the vector used for the transformation (Supplementary Figure S6).

## 3. Discussion

In this work, we successfully applied the CRISPR/Cas9 technology for the first time in chicory. CRISPR/Cas9 technology was already successfully used to knock out genes in other plants of the Asteraceae family such as salsify [40], lettuce [41] and dandelion [30]. Asteraceae as well as members of the Brassicaceae and Solanaceae are amenable to this biotechnological approach because of their high ability to regenerate [42]. Many successes in genome editing have also been achieved with plants belonging to the Poaceae family, not because of their great ability to regenerate but because they are certainly more studied.

The success of genome editing depends on the activity of the promoter used to drive the expression of the sgRNA. Arabidopsis *U6-26* promoter is frequently used in plants; however, in some plant species, such as in wheat and rice, it has been proven ineffective [43]. A study on soybean hairy roots (*Glycine max*) has also shown that endogenous *Gm-U6* promoter produced a mutation efficiency 2 to 6 times higher than that of Arabidopsis *U6-26* [13]. The chicory genome was screened for *U6* genes and 8 were identified based on their similarity with Arabidopsis *U6-26*. *CiU6-1*p was randomly selected among the six most conserved and was used to promote the expression of two sgRNAs targeting the fifth exon of the *CiPDS* gene. The *PDS* gene is often used as a target for the development of the method. Indeed, the mutant plants are albinos, which provides an efficient visual screen. Since 2013, it has been used in many species such as wheat [44], rice [43], populus [45], apple [46], watermelon [47], cassava [48] and banana [49,50]. In our study, the selection marker i.e., Basta resistance present on pYLCRISPR-sgRNA1-sgRNA2 was not used for selection of mutated plants. Nevertheless, this can be used to efficiently select transformants in other studies.

The CRISPR/Cas9 system was inserted in a unique vector and the *CiPDS* gene was knocked out by two different transformation methods: *A. rhizogenes* stable transformation and protoplast transient transformation with PEG. With both transformation methods, albino plants were regenerated, with mutation frequencies of 31.3% for *A. rhizogenes*-mediated transformation and 4.5% for protoplast transformation. The mutation frequencies obtained in other species are highly variable because of the intervention of numerous parameters, such as the ability of the species to be transformed, the transformation method used, the target design, the targeted gene. For example, mutation frequency in Arabidopsis *PDS* with PEG-protoplast transfection was 1.1% whereas in tobacco it was around 37% [1]. On the other hand, in soybean hairy roots the mutation frequency ranged from 14.7% to 20.2% depending on the mutated gene [13]. Many CRISPR/Cas9 studies have been summarized in [1]. In this study, the *A. rhizogenes*-mediated transformation has given a better mutation frequency (31.25%) than the protoplast transformation (4.5%). This better mutation rate may be due to the stable transformation, which allows the CRISPR/Cas9 system to continue the gene modification during hairy root development. This feature can give mosaic plants and if the target has some potential off-target sites, this can increase the unwanted mutations. Target design is a crucial step to avoid off-target cleavage in the genome. As a result, access to genome databases is very important for genome editing using CRISPR/Cas9 technology. However, not all plant species have complete or fully accessible genomic databases. Access to full genome sequence may facilitate the design of reliable targets for fine-tuning mutations. Nevertheless, it is essential and meaningful to confirm whether CRISPR/Cas9 can be applied, to gene function studies, in less studied plants.

Characterization of the chicory mutants resulted in the observation of mono- and biallelic mutants in plant regenerated from both kinds of transformation. In biallelic mutants, the ideal material for studying gene function, mutations were simultaneously detected at the desired positions in both targeted sites. This shows that it is possible to knock out several genes at the same time using both kinds of transformation methods in chicory. This multiplex genome editing is really convenient for gene function analysis, and with the *A. rhizogenes* transformation method, especially genes involved in specialized metabolism [51]. Indeed, in hairy roots, this metabolism is known to be very active.

In comparison with transformation and plant regeneration from protoplasts, it is quick and easy to establish hairy root lines in chicory. Plant regeneration is possible within 6 weeks after transformation and vernalization is not required for flowering [28,29]. Flowering occured as early as 1 week after regeneration and this can be a real advantage to study genes involved in the flower development or in reproduction. The drawbacks with using the *A. rhizogenes* method to produce CRISPR-mutant plants is that the T-DNAs are stably inserted into the genome. This is not a problem for many gene function analysis, but if the objective is to produce commercial varieties, it would be an inconvenient, because T-DNAs must be removed by self-pollination and this could be a long process in chicory. Indeed, chicory is a biennial plant and is often self-incompatible [52]. In this study, we showed that foreign DNA arising from the vector containing the CRISPR/Cas9 cassette can be inserted at a non-negligible frequency (3 plants out of 9 mutant plants) into the plant genome, after transient transformation in chicory. This phenomenon has also been shown in tobacco, where Cas9 DNA has been found in 17.2 % of mutant plants [53]. This could be caused by HR events promoted by the large number of plasmids present in the cells. In addition, once in the transfected cell, the plasmids are digested by endogenous nucleases and the resulting fragments could also be inserted inside the plant genome. This could explain the insertion of multiple vector DNA fragments at “on-target” site in the C8 mutant plant (Supplementary Figure S7). These results showed that after PEG-mediated transformation, it is extremely difficult to ascertain the location and the number of foreign DNA fragments inserted inside the plant genome. To obtain foreign DNA free plants would then be really challenging. To really set up a DNA free genome editing, it will be interesting to use pre-assembled ribonucleoprotein complexes (RNPs) instead of plasmid DNA to transform protoplasts in chicory. RNPs have been used in Arabidopsis, tobacco, lettuce, and rice protoplasts, through PEG-mediated transformation with the same conditions than when plasmid DNA are used, and the mutation frequencies seemed to be better [54]. Therefore, having regenerated mutant plants from protoplasts transformed with PEG-mediated transformation, is a very encouraging result for the use of RNPs in chicory.

In this study, we demonstrated that CRISPR/Cas9 technology could be used to generate mutant plants in chicory. Both methods of transformation tested were effective, but *A. rhizogenes*-mediated transformation was more efficient and faster. We showed that the CRISPR/cas9 system can be used to introduce small mutations at specific loci in the chicory genome. This method constitutes a powerful and easy-to-use tool for investigating gene function in chicory. To prevent foreign DNA integration into the genome that hinders the possibility of using this method to create commercial varieties with new agronomical traits, new editing systems such as RNPs could be assessed in chicory.

## 4. Materials and Methods

### 4.1. Plant Material

In vitro plants of *C. intybus* L. clone 17 and *C. intybus* L. var. Orchies were used in this study. Plants or seeds were provided by Florimond Desprez SA (Capelle-en-Pévèle, France). Seeds of *C. intybus* var. Orchies were surface sterilized and cultivated as described in Rambaud et al. [31]. Clone 17 plants were maintained on H10 solid medium (macro and micro-elements from Heller [55], Fe-Edta [56], 10 g/L sucrose, 6 g/L agar, pH 5.5) on a 16/8-h light/dark cycle with a temperature of 25 ± 2 °C in the light phase (37.6 ± 10.1 µmol/m^2^/s) and at 20 °C ± 2 °C in the dark phase. Clone 17 in vitro plants were subcultured every 8 weeks in H10 solid medium. The subcultures consisted to remove the old H10 solid medium, to cut the old leaves at around 1.5 cm of the basis of leaves, to carve it in V form to remove the roots and to insert the refreshed plants in H10 culture medium.

### 4.2. Sequence Identification and gRNA Design

The *CiPDS* and CiU6 promoter sequences were identified by searching the chicory genome (Unpublished database, Florimond Desprez SA, Cappelle-en-Pévèle, France) with the *Helianthus annuus* PDS (GeneBank accession AHA36971.1) and Arabidopsis *U6-26* genes, respectively. To mutate *CiPDS*, guide RNAs were designed based on their location in the gene and their GC content using the software crispor.tefor.net [35]. Guide1 and guide2 sequences were 5*′*-GGAACAATGAAATGCTGACG-3*′* and 5*′*-GCCTGCAATGTTAGGTGGAC-3*′*, respectively.

### 4.3. Vector Construction

This protocol is adapted from [36] and binary vector pYLCRISPR/Cas9P_35S_-B (Addgene plasmid #66190, Cambridge, MA, USA) was used as expression vector. The *C. intybus* U6-1 promoter (CiU6-1p) and the sgRNA scaffold were synthesized (IDT, Leuven, Belgium) (Supplementary Figure S8) and cloned into commercial plasmid pUCIDT-Kan resulting in the vector pKanCiU6-1p-sgRNAscaffold. The primers shown in Figure 2C have been synthetized by SIGMA-ALDRICH (Gillingham, UK). For guide adaptator hybridation, 1 µM of each primer was mixed in 0.5× Tris-EDTA buffer, pH 8.0 (10 µL) then heated to 90 °C for 10 s and returned to room temperature. To insert the target inside pKanCiU6-1p-sgRNAscaffold, 1.5 µL of cutsmart buffer (NEB, Ipswich, MA, USA), 100 ng of pKanCiU6-1p-sgRNAscaffold, 4.2 ng of target preparation, 10 U of BbsI (NEB, Ipswich, MA, USA), 40 U of T4 DNA ligase (NEB, Ipswich, MA, USA), and deionized water up to 15 µL were mixed and the digestion/ligation reaction was performed in a thermocycler using the following parameters: 3 cycles including 37 °C for 10 min, 10 °C for 5 min and 20 °C for 5 min and then 10 cycles including 37 °C for 10 min, 10 °C for 5 min, 20 °C for 10 min. Then *Escherichia coli* TOP 10 thermo-competent bacteria (In Vitrogen, Waltham, MA, USA) were transformed with 5 µL of the digestion/ligation product, and selected on LB agar plate containing 25 µg/mL kanamycin and 1 mM IPTG overnight at 37 °C. The colonies were picked and grown in 5 mL LB liquid medium supplemented with 25 µg/mL kanamycin, overnight at 37 °C. Plasmid was purified with the nucleospin plasmid purification kit (Macherey-Nagel, Düren, Germany). Two vectors, pKanCiU6-1p-sgRNA1 and pKanCiU6-1p-sgRNA2, containing the cassette “CiU6-1p-Guide-sgRNAscaffold” were obtained (Figure 2D). To add the *BsaI* restriction sites and the 4 nucleotides recombination sites for Golden Gate restriction/ligation method, at the ends of each cassette, PCR using a proofreading polymerase (PrimeStar HS DNA polymerase; TAKARA, Kasatsu, Japan) with specific primers were run: GG1-F ; GG1-R (Table A1): with pKanCiU6-1p-sgRNA1 and GG2-F ; GG2-R (Table A1) with pKanCiU6-1p-sgRNA2, as shown in Figure 2D. PCR products were purified with NucleoSpin Gel and PCR Clean-up kit (Macherey-Nagel, Düren, Germany) and were assembled with Golden Gate restriction/ligation method in pYLCRISPR/Cas9P_35S_-B as described by [36] to create pYLCRISPR-sgRNA1-sgRNA2 (Figure 2E).

### 4.4. A. rhizogenes Transformation

The *A. rhizogenes* strain 15834 (kindly provided by Marc Buée, INRA, Nancy, France) was transformed with pYLCRISPR-sgRNA1-sgRNA2 by electroporation, as described in Nagel et al. [57]. Following transfection, 950 µL of Yeast Extract Beef (YEB) solution were added in the cuvette, transferred to a 15 mL Falcon tube and incubated for 2–3 h at 28 °C at 200 rpm. After incubation, 100 µL were streaked on YEB agar plates with kanamycin (100 µg/mL) and incubated for 3 days at 28 °C.

### 4.5. Hairy Root Induction and Maintenance

Hairy root induction in *C. intybus* clone 17 using *A. rhizogenes* strain 15834 (Empty strain or transformed with pYLCRISPR-sgRNA1-sgRNA2) was performed on leaves, 7 days after subculture. The leaves were cut at petiole level and wounded with a scalpel previously soaked in a 3-day-old *A. rhizogenes* culture. The wounds were made perpendicularly to the central nervure, at 2 mm intervals, and the infected leaves were placed on Murashige and Scoog Hairy Roots (MSHR) solid medium (macro and micro-elements, Fe-Edta, vitamins from [56], 30 g/L sucrose, 6 g/L agar, pH 5.6) in Petri dishes, and grown at 25 °C ± 2 °C under 16 h light (37.6 ± 10.1 µmol/m^2^/s) and at 20 °C ± 2 °C under 8 h dark cycles. The first roots appeared after 12 to 13 days. Individual putative transgenic hairy roots of approximately 3 cm long, emerging from leaf explants transformed with *A. rhizogenes* were excised and immediately transferred to Petri dishes with MSHR solid medium containing antibiotic (ampicillin 300 mg/L). All the transformed roots were maintained separately as independent lines. The roots were subcultured every 7 to 10 days in new MSHR solid medium supplemented with antibiotics until the complete elimination of bacteria.

When the hairy root lines were found to be bacteria free, they were maintained in MSHR/2 solid medium (0.5 × macro and micro-elements, 0.5 × Fe-Edta, 0.5 × vitamins from [56], 30 g/L sucrose, 6 g/L agar, pH 5.6). When a hairy root line spontaneously made a shoot, it was transferred on H10 medium, for development and flowering. The hairy root lines, which did not regenerated spontaneously shoots after 4 weeks, were transferred to a C4 bud induction medium [38].

### 4.6. Protoplast Isolation, Transformation, and Culture

Leaves of chicory var. Orchies were cut from 12–14-day-old plants, the external epidermis was removed from each leaf and the leaves were incubated in the washing solution [38], added with 10 g/L cellulase Onozuka R-10 (Duchefa Biochemie, Haarlem, Nederland) and 5 g/L Macerozyme R-10 (Duchefa Biochemie, Haarlem, Nederland), overnight, in the dark, at room temperature. Then protoplasts were cleaned by filtration through a 50 µm steel mesh, collected, and washed three times with washing solution, by low speed centrifugation (150 g for 10 min). The protoplasts were resuspended in 10 mL MMG medium (100 mM MgCl_2_) [58]. Protoplasts were counted in Nageotte hemocytometer and incubated for 30 min on ice. After a low speed centrifugation (5 min at 150 g), MMG (100 mM MgCl_2_) was added to reach a final protoplast concentration of 6.10^5^ protoplasts/mL.

Thirty µg of pYLCRISPR-sgRNA1-sgRNA2 were added to 300 µL of protoplast solution and gently mixed. Then 330 µL of PEG4000 (30%, *m*/*v*) were added and mixed by gently tapping the tube. The transfection mixture was incubated at room temperature for 15 min. The transfection process was stopped by slowly adding 10 mL of washing medium. The diluted solution was centrifugated for 5 min at 150 g. The supernatant was removed, and 10 mL of MC1 culture medium [38] were added. Then, the protoplasts were partitioned in Petri dishes (3 mL per dishes) and incubated for 7 days at 30 °C in the dark. Callus proliferation and plant regeneration were achieved as described in [38].

### 4.7. DNA Extraction, Detection of Mutation, and CAS9 Amplification

Genomic DNA from calli, hairy roots and plants were extracted using the NucleoSpin Plant II kit (Macherey-Nagel, Düren, Germany) according to the manufacturer instructions. The genomic DNA extracted was used as a template to amplify the fifth exon of *CiPDS* by PCR. PCR was performed using a specific primer pair hybridizing between the two gRNAs (E5-F and E5-R) and the PCR products (around 420 bp) were separated in a 1% agarose gel. To analyze the mutations, the PCR products were sequenced with Sanger sequencing method using the same primers (E5-F and E5-R, Table A1) and the chromatograms were analyzed with CodonCodeAligner software [39]. The *CAS9* was amplified with specific primer pair (C9-F and C9-R, Table A1) and PCR products (around 410 bp) were separated in a 1% agarose gel. For the analysis of the hairy root lines, control assays were carried with DNA extracted from plants transformed with the wild-type *A. rhizogenes* strain 15834.

## Figures and Tables

**Table 1:** Mutation analysis in the *CiPDS* gene. Analysis were performed on plants regenerated from protoplasts or hairy roots.

				Target 1						Target 2					
Transformation type	N° of lines	N° of albino lines	Mutation frequency (%)	Biallelic mutation			Mono allelic mutation	No mutation	Mutation efficiency	Biallelic mutation		Mono allelic mutation	No mutation	Mutation efficiency	
				HM^1^	HZ^2^	%				HM^1^	HZ^2^				%
Protoplast	198	9	4.5	2	3	55	1	3	66.6%	5	4	100	0	0	100%
Hairy root	32	10	31.3	1	5	60	3	1	90.0%	1	7	80	1	1	90%

^1^ HM: homozygous. ^2^ HZ: heterozygous.

## Supplementary Materials

Supplementary materials can be found at https://www.mdpi.com/1422-0067/20/5/1155/s1.

## Appendix A

1) Used software “crispor-tefor.net” [35] and “codoncode.com” [39].
2) Accession numbers: U6-1: MK455772, U6-2: MK455773, U6-3: MK455774, U6-4: MK455775, U6-5: MK455776), U6-6: MK455777, U6-7: MK455778, U6-8: MK455779, CiPDS: MK455771, HaPDS: AHA36971.1.

## Appendix B

**Table A1:** Sequences of primers used in this study.

Name	Sequences (5′-3′)
Forward primer (guide1)	ATCGGAACAATGAAATGCTGACG
Reverse primer (guide1)	AAACCGTCAGCATTTCATTGTTC
Forward primer (guide2)	ATCGCCTGCAATGTTAGGTGGAC
Reverse primer (guide2)	AAACGTCCACCTAACATTGCAGG
GG1-F	ATATATGGTCTCACTCGAAAGAACCAACCTGTTTTCATAGC
GG1-R	ATTATTGGTCTCAACCTAAAAAAAGCACCGACTCGGTG
GG2-R	ATATATGGTCTCAAGGTAAAGAACCAACCTGTTTTCATAGC
GG2-F	ATTATTGGTCTCAACCGAAAAAAAGCACCGACTCGGTG
E5-F	TTTCTTGAAGTTGGAGCTTACC
E5-R	AAACCTCAGTAGTAACTCGATCTG
C9-F	AAGCACGTTGCTCAGATCCT
C9-R	CCGTTCGTCTCGATAAGAGG
