# Supplementary material for: Efficient Genome Editing Using CRISPR/Cas9 Technology in Chicory
Source: Int J Mol Sci. 2019 Mar 6;20(5):1155. doi: 10.3390/ijms20051155 (PMC6429391; doi:10.3390/ijms20051155)
Supplement: Supplementary file 1 [file ijms-20-01155-s001.pdf]

ttaggatattcggtgaggtgatatgtctctgtttggaaatgtctccgccattaactcaag
tggaaagtgtatagtaatgaatctttcaagcacacagatcacttcaaaagactgtttcaa
catcacctcaggacaaaaagatgtactctcatttggatgctgtgatgccatgggtcacag E1
attgcaattcccaagtgcccgttcttttacaccaaatcaaagaagaatatctcccttt
aaaggtacctttatatgcttctaattattgttcatagtatcaagttaatgaacaaatgta
tctctaataatcaaatagtagtttgtgttgactatccaagaccagatcttgacaacacc
tctaatttcttggaagctgcttacttgtcttcaaccttcacaacttcccctcgcccattt E2
aagccattgaagattgtaattgctggtgcaggtaaaattaaaaacttaattttaaatt
tattttttactaaccttactttgattatctgttatcaaactaacttttcaaattttgca
ttttcaggtttggctggtttatcaactgcaaagtatttggctgatgcaggtcacaagcca E3
atttgttagaagcaagagacgttcttggtggaaaggtttataaactataaagtttctat
ctttattttcattttatctcaaatacatttctcttattggccttctaaattattacatag
gtggcagcttggaaagatgatgatggagattggtacgagacaggtttacacatattctgt E4
aagttttaaccttttttattatccttcaagtttctaaaaatatagtctttttttggatacc

E5-F

taatgctgaatgaactttttaattttcttgaagttggagcttacccaaatatacagaatt
tgtttggagagttaggaattaatgatagattgcaatggaaggaacattctatgatatttg
caatgccaaataagcctggagaatttagtcggtttgactttccagatgttttgcctgcac
cattaaatggaattttttgctatattgaggaacaatgaaatgctgacg**tgg**cctgaaaaag E5
taaaatttgcaattgggcttttgcctgcaatgttaggtggac**agg**cttatgttgaggccc
aagatggtttgagtgttcaagactggatgagaaaacaagtatgtaaatgtaatcattaaa
gtaatattttttatctaaaactttagataattagattactaatcaatcttttttaattttttt
tagggcatacсagatcgagttactactgaggtttttattgccatgtcaaaggcattaaac

E5-R

tttataaatccagatgaactttcaatgcagtgtattctcattgctctcaaccggtttctt E6
caggtaaaattttttattaataatccggaagaaacaatatttttttttttcaattattgttg
ttgattaatataaaataaatcaacaggagaaacatggttccaagatggcatttttagatg
gaagcccacccgaaagactttgccagccaattgttgaccatattgagtcacttggtggcc E7
aagtcagactcaattcaagaatacaaaaaattgaattaaacaaagacggaactgtccgga
actttctactgaatgacgggaatgttctagaagctgatgcttatgtattcgccacccccg
gtatgtttcactatttatatttatctctatagtacatattcttgatttctagggtccaaa
ttattgaattattttactttttttttttgtgtataaaacagttgacattttaaagcttctt E8
ttacctgaagaatggaaaccaattccatatttcaaaaagttagagaaactagtcggtgtt
ccggttataaacgttcatatatggttagtcgttgacttcttgactttgactttgacttta
atcataaccgttttgtattaatctttaaaattattttcaggtttgataggaaactgaaaa
acacatatgatcacctactattcagtaggtcgcctcttttgagtgtatatgctgacatgt
cagtgacatgtaaggaatattatgatccgaatcggtcaatgttggaattggttttttgcac
ctgcagaagaatggatttcaagaagtgacactgatattattgatgcaacaatgagtgaac
tttcaaggcttttttcctgatgaaatttcagcagatggaagtaaagcaaaaatcctgaaat E9
ataaagttgttaaaacaccaagatctgtttataaaaccataccagattgtgaaccttgta
gaccactacaaagatctccaattgaggattttatttagctggtgattatactaaacaga
agtatttagcttcaatggagggtgcggttttatctggaaagttttgtgcacaagctattg
taaaggtaagttttaagtatataaaagtaggttgttataattgttaattggttaatgatt
tttggattttgtttttttaggattatgagttgcttgctgcacgtaaggagtggttgctga E10
ggcaagccttgtgtaagttaataattaatggatgaattttaaggagctcttacaagttca

Supplementary Figure S1. *CiPDS* DNA sequence, sgRNA target sites and the primer sites for PCR amplification of the fifth exon. The exons are depicted in red and introns in dark. Blue nucleotides indicate the target sites, bold red nucleotides indicate the PAM sequences. Nucleotides highlighted in blue indicate start and stop codons, respectively.

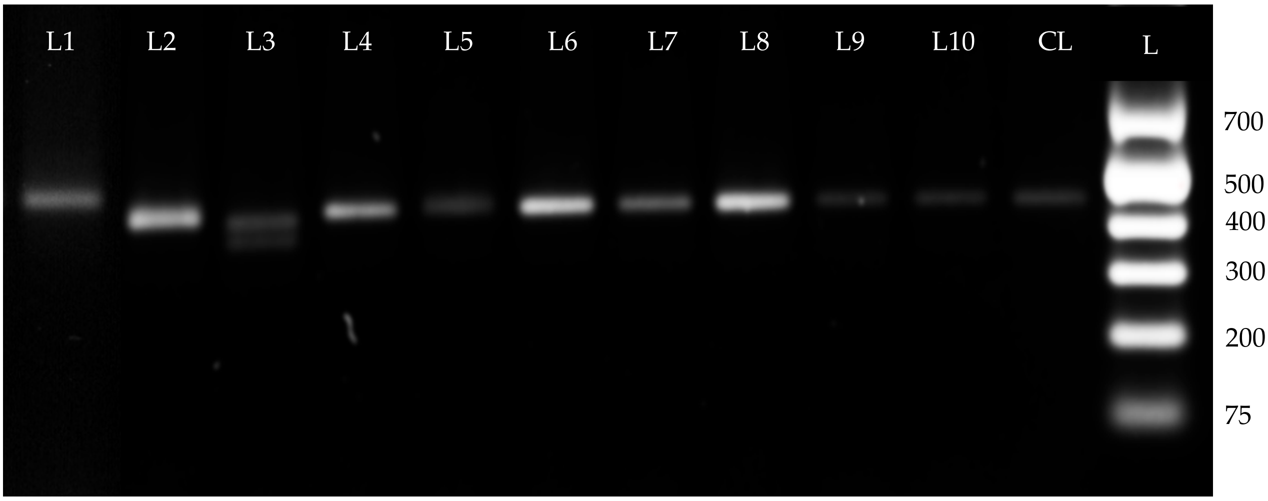

Supplementary Figure S2: DNA amplification of albino plant regenerated from hairy root lines by PCR with specific primer pair (E5-F and E5-R) surrounding the two target sites in the fifth exon of the *CiPDS*. L = Line, CL = Control Line (DNA from plant transformed with the wild type *A.rhizogenes* strain 15834), L = Ladder.

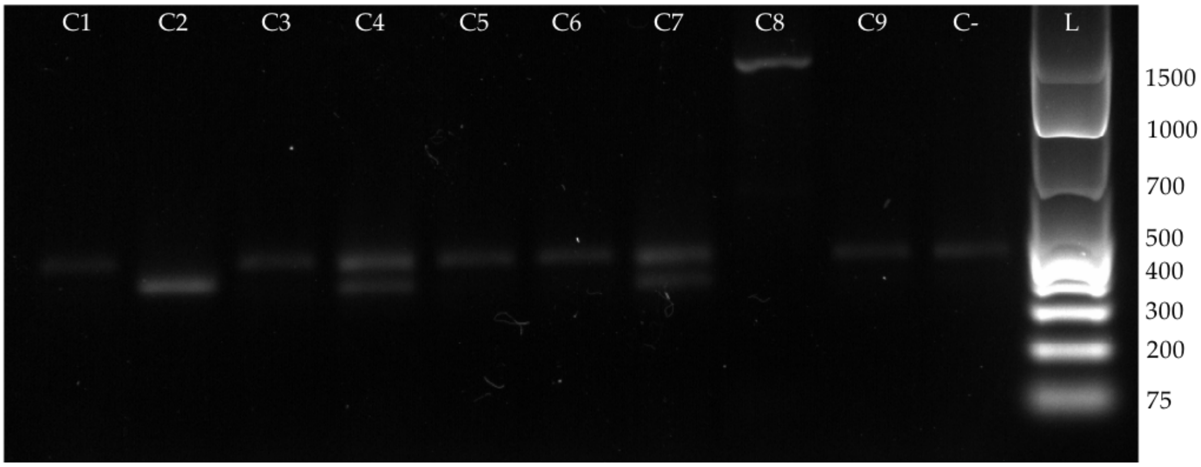

Supplementary Figure S3: Amplification by PCR of DNA extracted from albino plants regenerated from calli with specific primer pair (E5-F and E5-R) surrounding the two target sites in the fifth exon of the *CiPDS*. C = Callus, C- = DNA from plant regenerated from non-mutated callus, L = Ladder.

WT-KPGEFSRFDFPDVLPAPLNGIFAILRNNEMLTWPEKVKFAIGLLPAMLGGQAYVEAQDGL...
L1.1 - KPGEFSRFDFPDVLPAPLNGIFAILRNNENVA*
L1.2 - KPGEFSRFDFPDVLPAPLNGIFAILRNNEMLRGLKK*
L2.1 - KPGEFSRFDFPDVLPAPLNGIFAILRNNEMRRGLKK*
L2.2 - KPGEFSRFDFPDVLPAPLNGIFAILRNNEMLVA*
L3.1 - KPGEFSRFDFPDVLPAPLNGIFAILRNNEMLRGLKK*
L3.2 - KPGEFSRFDFPDVLPAPLNGIFAILRNNEMLTGLC*
L4.1 - KPGEFSRFDFPDVLPAPLNGIFAILRNNEMLTWPEKVKFAIGLLPAMLGVTGLC*
L4.2 - KPGEFSRFDFPDVLPAPLNGIFAILRNNEMHVA*
L5.1 (R) - KPGEFSRFDFPDVLPAPLNGIFAILRNNEMLTWPEKVKFAIGLLPAMLGGQAYVEAQDGL...
L5.2 (R) - KPGEFSRFDFPDVLPAPLNGIFAILRNNEMLTWPEKVKFAIGLLPAMLGGTGLC*
L5.1 (P) - KPGEFSRFDFPDVLPAPLNGIFAILRNNEMLTWPEKVKFAIGLLPAMLGVTGLC*
L5.2 (P) - KPGEFSRFDFPDVLPAPLNGIFAILRNNETWPEKVKFAIGLLPAMLGVDRLMLRPKMV*
L6.1 - KPGEFSRFDFPDVLPAPLNGIFAILRNNEM-TWPEKVKFAIGLLPAMLGVTGLC*
L6.2 - KPGEFSRFDFPDVLPAPLNGIFAILRNNEMLRGLKK*
L7.1 - KPGEFSRFDFPDVLPAPLNGIFAILRNNEMRRGLKK*
L7.2 - KPGEFSRFDFPDVLPAPLNGIFAILRNNEMLVA*
L8.1 - KPGEFSRFDFPDVLPAPLNGIFAILRNNEMLTWPEKVKFAIGLLPAMLGDRLMLRPKMV*
L8.2 - KPGEFSRFDFPDVLPAPLNGIFAILRNNEMLTWPEKVKFAIGLLPAMLGETGLC*
L9.1 - KPGEFSRFDFPDVLPAPLNGIFAILRNNEMLTWPEKVKFAIGLLPAMLGRTGLC*
L9.2 - KPGEFSRFDFPDVLPAPLNGIFAILRNNEMLRGLKK*
L10.1- KPGEFSRFDFPDVLPAPLNGIFAILRNNEMLRGLKK*
L10.2- KPGEFSRFDFPDVLPAPLNGIFAILRNNEMLRGLKK*

Supplementary Figure S4: Predicted amino acid sequences of the CiPDS from the 10 albino hairy root lines and their shoots. Lx.x = Line x. allele x. For L5 (R) = Hairy root stage sequence, (P) = Plant stage sequence. In blue, the amino acids that differ from the wild type sequences. (-) shows the deleted amino acids and (*) shows the end of the amino acid sequence.

WT- KPGEFSRFDFPDVLPAPLNGIFAILRNNEMLTWPEKVKFAIGLLPAMLGGQAYVEAQDGL...
C1.1 - KPGEFSRFDFPDVLPAPLNGIFAILRNNEMLTWPEKVKFAIGLLPAMLGVTGLC*
C1.2 - KPGEFSRFDFPDVLPAPLNGIFAILRNNEMLTWPEKVKFAIGLLPAMLGVTGLC*
C2.1 - KPGEFSRFDFPDVLPAPLNGIFAILRNNETGLC*
C2.2 - KPGEFSRFDFPDVLPAPLNGIFAILRNNETGLC*
C3.1 - KPGEFSRFDFPDVLPAPLNGIFAILRNNEMLRGLKK*
C3.2 - KPGEFSRFDFPDVLPAPLNGIFAILRNNEMLYVA*
C4.1 - KPGEFSRFDFPDVLPAPLNGIFAILRNNDVA*
C4.2 - KPGEFSRFDFPDVLPAPLNGIFAILRNK -AYVEAQDGL...
C5.1 - KPGEFSRFDFPDVLPAPLNGIFAILRNNEMLTWPEKVKFAIGLLPAMLGTGLC*
C5.2 - KPGEFSRFDFPDVLPAPLNGIFAILRNNEMLTWPEKVKFAIGLLPAMLGETGLC*
C6.1 - KPGEFSRFDFPDVLPAPLNGIFAILRNNEMLRGLKK*
C6.2 - KPGEFSRFDFPDVLPAPLNGIFAILRNNEMLTWPEKVKFAIGLLPAMLGGTGLC*
C7.1 - KPGEFSRFDFPDVLPAPLNGIFAILRNNERGLKK*
C7.2 - KPGEFSRFDFPDVLPAPLNGIFAILRNK -AYVEAQDGL...
C8.1 - KPGEFSRFDFPDVLPAPLNGIFAILRNNEMLRGLKK*
C8.2 - KPGEFSRFDFPDVLPAPLNGIFAILRNNEMLRGLKK*
C9.1 - KPGEFSRFDFPDVLPAPLNGIFAILRNNEMLTWPEKVKFAIGLLPAMLGGTGLC*
C9.2 - KPGEFSRFDFPDVLPAPLNGIFAILRNNEMLTWPEKVKFAIGLLPAMLGGTGLC*

Supplementary Figure S5: Predicted amino acid sequence of the CiPDS from the 9 albino calli and their shoots. Cx.x = Callus x. allele x. In blue, the amino acids that differ from the wild type sequences, (-) shows the deleted amino acids and (*) shows the end of the amino acid sequence.

(TGAGTAGTTCCCAGATAAGGGAATTAGGGTTCTTATAGGGTTTCGCTCATGTGTTGAGCATATAAGAAACCCTTAGTATGTATT
TGTATTTGTAAAATACTTCTATCAATAAAATTTCTAATTCCTAAAACCAAAATCCAGTGACGCGGCCGCATAAGGCCGCCATCTC
GATGACGATGTTCTCAGGCTTGTGACGACCCGTAAGCGGCTGGGTTGCCTGCCGGCCCTGCAATGGCACTGGAACCCCCAAGC
CCGAGGAATCGGCGTGAGCGGTCGCAAACCATCCGGCCCGGTACAAATCGGCGCGGCGCTGGGTGATGACCTGGTGGAGAA
GTTGAAGGCCGCGCAGGCCGCCCAGCGGCAACGCATCGAGGCAGAAGCACGCCCCGGTGAATCGTGGCAAGCGGCCGCTGA
TCGAATTGCGCAACCGGAACCCGTACATTGGGAA(nnn...nnn)TTCCCAATGTACGTGCTATCCACAGGAAAGAGAACTTTTCG
ACCTTTTTCCCCTGCTAGGGCAATTTGCCCTAGCATCTGCTCCGTACATTAGGAACCGGCGGATGCTTCGCCCTCGATCAGGTTG
CGGTAGCGCATGACTAGGATCGGGCCAGC)

Supplementary Figure S6: Partial sequence of the DNA fragments inserted at the target 2 site of the C8 mutant. The insert is composed of more than 6 fragments (depicted in red, black, green, purple or orange) of the pYLCRISPR-sgRNA1-sgRNA2. (nnn…nnn) is a part of the fragment, which was not sequenced.

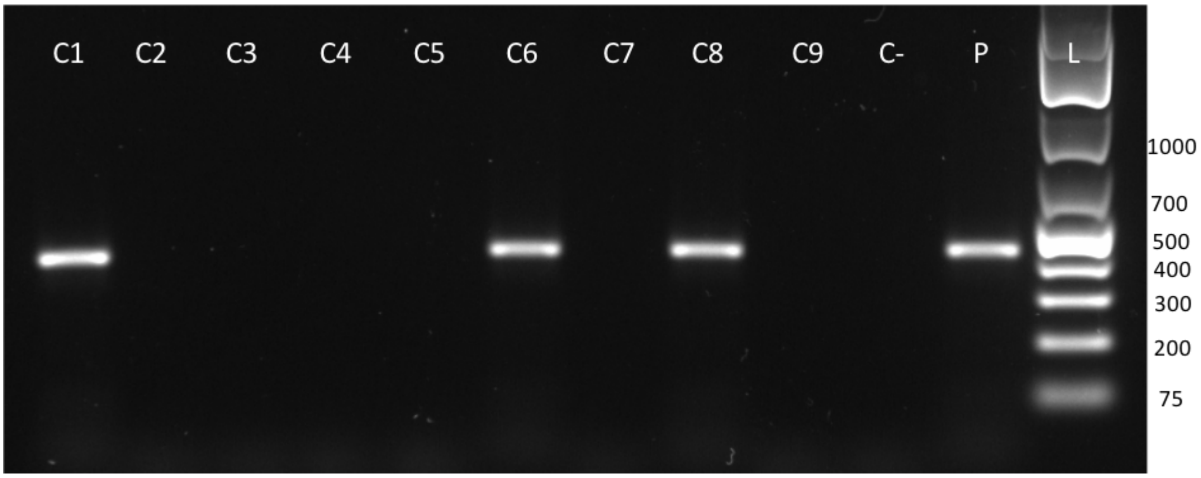

Supplementary Figure S7: DNA amplification of albino plants regenerated from calli by PCR with specific primer pair (C9-F and C9-R) hybridizing in Cas9 gene. C = Callus, C- = DNA from plant regenerated from non-mutated callus, P = Plasmid pYLCRISPR-sgRNA1-sgRNA2 DNA, L = Ladder.

GGAATTCGGCTGGTCTCTCTCGAAAGAACCAACCTGTTTTCATAGCTCTGTTGACTGATAGAGTTTTGATA
TGTTCAACTTTCAGGTAAAACCAAGAGGATAGTAAAATTGAGGGTTAGCTTACAAATATTTATCGGTTCCTA
GCTATCGTAATGCCATGGCTTCCCACATCGCTCATTGGAGCAACATCGCCATGCTTTATATAGCCTCGCCT
CCAACCATTT**ATCG**ATGTCTTCgAGTTAGCTCACTCATTAGGCACCCCAGGCTTTACACTTTATGCTTCCG
GCTCGTATGTTGTGTGGAATTGTGAGCGGATAACAATTTCACACAGAAACAGCTATGACCATGATTACGC
CAAGCTATTTAGGTGACACTATAGAATACTCAAGCTATGCATCAAGCTCAATGGGTCTAGTCTGTAGATAC
CCATCACACTGGCGACCGCTCGAACATCAGTTTAAGGTTTACACCTATAAAAGAGAGAGCCGTTATCGTC
TGTTTGTGGATGTACAGAGTGATATTATTGACACGCCGGGGCGACGGATGGTGATCCCCCTGGCCAGTG
CACGTCTGCTGTCAGATAAAGTCTCCCGTGAACTTTACCCGGTGGTGCATATCGGGGATGGAAGCTGGC
GCATGATGACCACCGATATGGCCAGTGTGCCTGTCTCCGTTATCGGGGAAGAAGTGGCTGATCTCAGCC
ACCGCGAAAATGACATCAAAAACGCCATTAACCTGATGTTCTGGGGAATATAAATGTCAGGCCTGAATGG
CGAATGGACGCGCCCTGTAGCGGCGCATTAAGCGCGGCGGGTGAGCGTgGAAGACTA**GTTT**TAGAGCTA
GAAATAGCAAGTTAAAATAAGGCTAGTCCGTTATCAACTTGAAAAAGTGGCACCGAGTCGGTGCTTTTTTT
TGAGACCGACCGGATCCC

Supplementary Figure S8: Sequence of the cassette "CiU6-PlacZ ccdb-sgRNAscaffold" harboring by the plasmid pKanCiU6-1p-sgRNAscaffold. The CiU6-1p, the selectable marker PlacZ-*ccdB* and the sgRNA scaffold are written in blue, yellow and green respectively. In red is highlighted the *BbsI* recognition site which achieves a cleavage on the left side (shown in underlined and bold). Note that the G present in the 4 nucleotides of the cutting site, is the transcription initiator. Highlighted in blue is the *BbsI* recognition site which achieves a cleavage on the right side (shown in underlined and bold).
